# 

**Published:** 1917-08

**Authors:** 


### ANNOUNCEMENTS

---

AMERICAN SOCIETY OF ORTHODONTISTS, Excelsior Springs, Mo., Sept. 5-8, 1917. Secretary, Dr. F. M. Casto, Rose Bldg., Cleveland, Ohio.

---

NATIONAL DENTAL ASSOCIATION, New York City, October 22, 23, 24, 25, 26, 1917. Secretary, Dr. Otto U. King, Huntington, Ind. Program in next issue.

---

NATIONAL ASSOCIATION OF DENTAL FACULTIES, New York City, N. Y., October 19, 20, 1917. Secretary, Dr. C. C. Allen, Kansas City, Mo.

### NATIONAL DENTAL LEGISLATION

COLUMBUS, OHIO, July 10, 1917.

*My Dear Doctor:*

I think it has been generally understood that the Legislative Committee, National Dental Association, has had for some time some agreed-upon legislation ready for introduction in Congress, but has been waiting for what would seem to be a favorable opportunity. I say a favorable opportunity, since everyone can not fail to recognize that Congress has been devoting its time principally to emergency measures for the past few months and this was no time to introduce legislation such as ours, especially when it is remembered that approximately only one year has elapsed since two additional grades for both the Army and Navy Dental Corps were granted. Also, provision was made for a Dental Reserve Corps in the army and improvement secured for the Reserve Corps of the navy, by making provision for promotion of the members of the Navy Reserve Corps, the same as in the Regular Corps, when called into active service. We also secured the same provision for promotion for the Navy Medical Reserve Corps.

It may be interesting to know at this time that at the time our army legislation, as passed by the senate and house last year, was pending in the conference committee, we prepared a substitute along lines developed in the discussion when the Pomerene amendment was before the senate for consideration. At that time a number of senators expressed themselves as willing to give the Dental Corps the same status as the Medical Corps. However, under the limitation of the action of the senate and house we necessarily limited our grades in the proposed substitute, to that of Major, as that was the highest grade which had been granted by either branch of Congress. This substitute was not accepted, but it may be said that the following amendment by Senator Lodge, introduced

July 3, is more or less the direct result of that proposition:

"Intended to be proposed by Mr. Lodge, to the bill (H. R. 4897) to amend section ten of the national defense Act approved June third, nineteen hundred sixteen, and for other purposes," as follows:

*"Hereafter the Dental Corps shall consist of commissioned officers of the same grades and proportionally distributed among such grades as are now or may hereafter be provided by law for the Medical Corps, who shall have the rank, pay and allowances of the officers of corresponding grades in the Medical Corps, including the right to retirement as in the case of other officers, and there shall be one dental officer for every one thousand of the total strength of the Regular Army, authorized from time to time by law:*

*PROVIDED, That all laws relating to the examination of officers of the Medical Corps for promotion shall be applicable to officers of the Dental Corps:*

*PROVIDED FURTHER, That Dental Examining and Review Boards shall consist of one officer of the Medical Corps and two officers of the Dental Corps:*

*AND PROVIDED FURTHER, That immediately following the approval of this act all Dental Surgeons then in active service shall be recommissioned in the Dental Corps in the grades herein authorized in the order of their seniority and without loss of relative rank in the Army:*

*AND PROVIDED FURTHER, That First Lieutenants in the Medical Department shall be promoted to the grade of captain upon the completion of three years' service in that grade in the Medical Department and upon passing the examinations prescribed by the President for promotion."*

In order that the connection between the Lodge Amendment and H. R. 4897 may be fully understood the latter is herewith incorporated: "PROVIDED, That during the existing emergency Lieutenants in the Medical Corps of the Regular Army and of the National Guard shall be eligible to promotion as captain upon such examination as may be prescribed by the Secretary of War."

I will state that this was introduced by Mr. Dent, Chairman of the House Military Committee, and passed the House of Representatives June 25. It is now before the Senate Military Affairs Committee for consideration, where the Lodge Amendment was also referred. The Food Bill has been occupying the attention of the Senate for some time; Senator Chamberlain has active charge of it and he is also Chairman of the Senate Committee on Military Affairs. The final vote on the Food Bill is scheduled for July 21 and then it would seem that such emergency or necessary legislation as may be required will receive fairly prompt attention. Further, Congress will probably adjourn soon and for these reasons whatever support is to be given our proposed legislation must necessarily be done immediately. Drs. L. L. Barber, W. H. G. Logan and myself spent Saturday, July 7, in Washington and conferred with a number of friends interested in seeing that both the Regular and Reserve Corps are placed on a proper status, as compared with other professional Corps and the importance of the services rendered. This proposed legislation will automatically give three grades to the Dental Reserve Corps members, that is, First Lieutenants, Captains and Majors.

The needs of the War will call many of our members into the Reserve Corps and you are undoubtedly interested in doing your part both in rendering service and cooperating in promoting this legislation. In order for this cooperation to be effective prompt action will be necessary.

You can very positively assist in this legislation by writing immediately, or more preferably using Night Letter telegrams, to your senators respectfully soliciting their support of the Lodge Amendment to House Bill 4897

and request them to see Senator Chamberlain and other members of his committee in behalf of the Lodge Amendment. You can very properly emphasize the fact that the Europeon War has clearly demonstrated the importance of the cooperation of the Medical and Dental profession in treating many of the wounds resulting from present-day warfare and that there is no reason why our representatives in the Federal service should not be accorded the same status as the members of the Medical Corps.

The following is a list of the Senate Committee on Military Affairs and if your senator is on this Committee you are in a position to render most valuable assistance:

*Senate Committee on Military Affairs:* George E. Chamberlain, Chairman, of Oregon; Gilbert M. Hitchcock, of Nebraska; Duncan U. Fletcher, of Florida; Henry L. Meyers, of Montana; Charles S. Thomas, of Colorado; Morris Sheppard, of Texas; J. C. W. Beekman, of Kentucky; William F. Kirby, of Arkansas; James A. Reed, of Missouri; Kenneth McKellar, of Tennessee; Francis E. Warren, of Wyoming; James H. Brady, of Idaho; John W. Weeks, of Massachusetts; James W. Wadsworth, Jr., of New York; Howard Sutherland, of West Virginia; Harry S. New, of Indiana; Joseph S. Frelinghuysen, of New Jersey.

Now is the time to pull together and as chairman of the Legislative Committee I will appreciate it if those replies which are either strongly favorable or unfavorable to this legislation will be forwarded to me. Those merely acknowledging receipt of letter, etc., will not be of any service to our cause.

Thanking you in advance for your prompt assistance, I remain, fraternally yours, HOMER C. BROWN, Chairman, Legislative Committee, N. D. A., 609 Hartman Bldg., Columbus, Ohio.

